# Leakages suppression by isolating the desired quantum levels for high-temperature terahertz quantum cascade lasers

**DOI:** 10.1038/s41598-021-02301-3

**Published:** 2021-12-08

**Authors:** Li Wang, Tsung-Tse Lin, Mingxi Chen, Ke Wang, Hideki Hirayama

**Affiliations:** 1 THz Quantum Device Team, RIKEN Center for Advanced Photonics, 519-1399 Aramaki-aza Aoba, Aoba-ku, Sendai, 980-0845 Japan; 2 School of Electronics Science and Engineering, Nanjing University, 163 Xianlin Street, Qixia District, Nanjing, 210046 China

**Keywords:** Optics and photonics, Lasers, LEDs and light sources, Semiconductor lasers

## Abstract

The key challenge for terahertz quantum cascade lasers (THz-QCLs) is to make it operating at room-temperature. The suppression of thermally activated leakages via high lying quantum levels is emphasized recently. In this study, we employ the advanced self-consistent method of non-equilibrium Green’s function, aiming to reveal those kinds of leakages in the commonly used THz-QCL designs based on 2-, 3- and 4-quantum well. At the high temperature of 300 K, if all the confined high lying quantum levels and also the continuums are included within three neighboring periods, leakages indeed possess high fraction of the total current (21%, 30%, 50% for 2-, 3- and 4-quantum well designs, respectively). Ministep concept is introduced to weaken those leakage channels by isolating the desired levels from high lying ones, thus the leakages are well suppressed, with corresponding fractions less than 5% for all three designs.

## Introduction

Terahertz (THz) spectral region (300 GHz–10 THz) remains at low level of development, to a great extent, the lack of compact, solid-state, coherent radiation source is a primary cause. In fact, extensive applications have been carried out in fields from imaging and chemical sensing to telecommunications^1–3^, demonstrating the high potentials of THz wave. THz quantum cascade lasers (THz-QCLs) are treated as the most promising candidate for solid-state THz radiation source^4^. This type of lasers relies on intersubband transition between confined quantum levels within stacked quantum wells (QWs) structure. With a delicate balancing of the injection and extraction process, population inversion between the two laser levels can be fulfilled. The most used optical gain medium for THz-QCLs is gallium arsenide related quantum structures (quantum wells/barriers: GaAs/AlGaAs), thanks to its well-established growth technique of molecular beam epitaxy (MBE) which can ensure an atomic-scale tailoring of QWs structure. However, the main limitation of THz-QCLs is that its operation requires additional cooling component.

Since the first success of THz-QCLs in year 2002^5^, significant efforts have been devoted to raise the maximum operating temperature (*T*_max_). The dominant mechanism of thermally degradation was initially ascribed to a nonradiative channel between two laser levels^6,7^, that refers to hot electrons in the upper laser level gaining enough kinetic energy, then relax down to the lower laser level via longitudinal optical (LO) phonon emission (a nonradiative manner instead of THz photon emission (radiative manner)). In the early designs of THz-QCLs^8,9^, upper/lower laser levels are normally coupled with a strong transition dipole moment (also named as vertical transition), easily activating this thermally nonradiative channel mentioned above. The corresponding strategy is to make the transition from vertical to diagonal by reducing the spatial overlap of upper/lower laser levels. The validity of this scheme is well clarified in experiments^10–12^. At the same time, thermal backfilling mechanism is also paid attentions in which electrons can be scattered back to the lower laser level and directly reduce the population inversion^13,14^. This mechanism is quite sensitive to temperature specifically in the design of bound- to-continuum^9^ mainly because the energy spacing between injection area and laser levels is small. The development of LO-phonon assisting depopulation scheme effectively relaxes this limit.

The very recent breakthrough of *T*_max_ at 250 K emphasizes the importance of leakages engineering^15^. It is relatively difficult to make a quantification of thermally activated leakages in QCLs due to the lack of direct measurements. In fact, the leakages issue was extensively studied in mid-infrared QCLs relying on indirect measurement ways^16,17^. Some previous studies also point out its possible effects on the performance of THz-QCLs^18^, but mainly focus on the leakages flowing over low-barrier into the continuum. Recent systematic investigation in tall-barrier designs (Al% in AlGaAs barrier: 15% →  > 20%)^19–22^ offers new insights regarding those thermally activated leakages. Tall barrier basically can create deep well to confine more high lying quantum levels. Those levels may exist as parasitic channels which can be easily activated when the temperature increases, as a result, reduces the lifetime of upper laser level. However, different view is also shown on the roles of those high lying levels^23^. Therefore, it still urgently continue to clarify the leakages in THz-QCLs especially under 300K operation.

Here we emphasize two considerations: (1) In previous study, the models describing the quantum transport in THz-QCLs generally assume a simple *n*-level system, in which the upper laser level is positioned highest in energy, where *n* is the number of desired quantum levels in each period. When more levels are included, the transports may be disturbed as those levels may create some parasitic channels (especially at high temperature). Furthermore, the loss of coherence is also important to estimate long-distance leakages (for example, leakages across neighboring periods). Here, we introduce the non-equilibrium Green’s functions (NEGF) running within a self-consistent manner. The number of quantum levels including in the calculation can be freely selected just by tuning the cut-off energy range, and the coherence/decoherence are originally considered; (2) It is no doubt that the leakages should be discussed based on detailed designs. In this work, three commonly used designs for high temperature operation are compared.

As show in the main content, even with tall barrier (at least, Al% of 25% in AlGaAs), the thermally activated leakages out of the active region are very strong in all three designs, even partly escape over the tall barrier. The high lying levels absolutely play critical roles. The magnitude of leakages depends on the design itself. By introducing ministep into the widest QW, the desired levels can be isolated by moving upward the high lying levels, thus well suppressing the leakages. This concept is valid for different designs.

## Results and discussions

**Figure 1:**
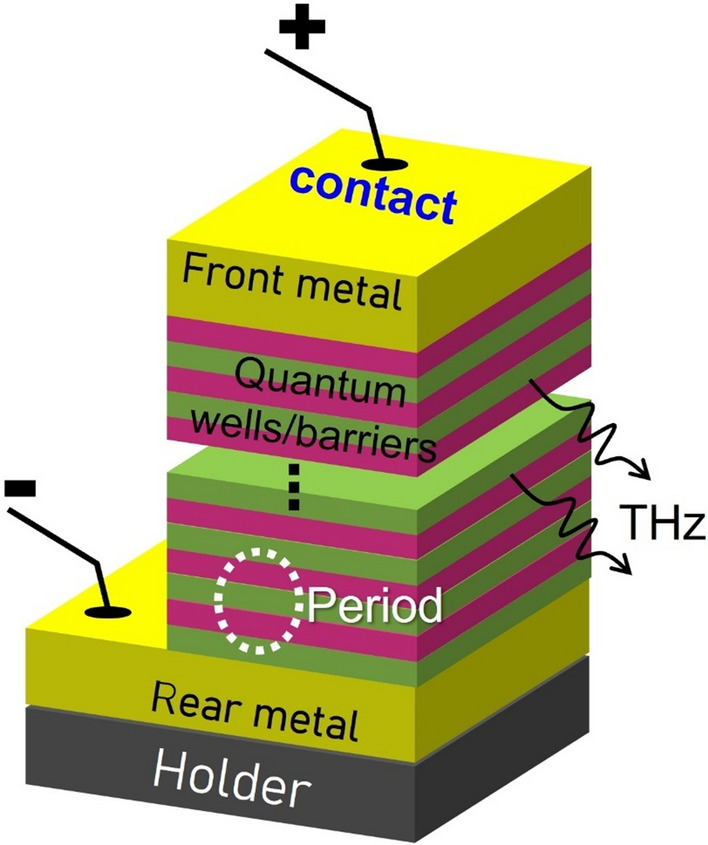
Schematic diagram of the terahertz quantum cascade lasers. The lasing behavior relies on hundreds of periods containing thousands of quantum wells/barriers (GaAs/AlGaAs). A double-metal waveguide structure is shown for side emission of THz wave.

A key improvement in THz-QCLs design is to depopulate the lower laser level assisting by LO phonon emission. Based on this experience, the main designs presently are summarized as schemes of three-level direct-phonon (based on 2-QW), four-level resonant-phonon (based on 3-QW) and five-level bound-to-continuum (based on 4-QW). In this work, the leakages are investigated based on those three designs. The schematic diagram of THz-QCLs is shown in Fig. 1. Hundreds of periods are stacked together and each of them consists of multiple quantum wells (GaAs) and barriers (AlGaAs). Electrons are pumped into the active region from cathode electrode, perform quantum transport perpendicular the QWs. In each cascaded period, electrons can donate THz photons until they are finally extracted out through anode electrode. Therefore, a key feature of QCLs is, the photons are times of electrons simply depending on the number of stacked periods. A total 10 μm-thick QWs is essential, and the double-metal waveguide is used for achieving high-temperature operations.

**Figure 2:**
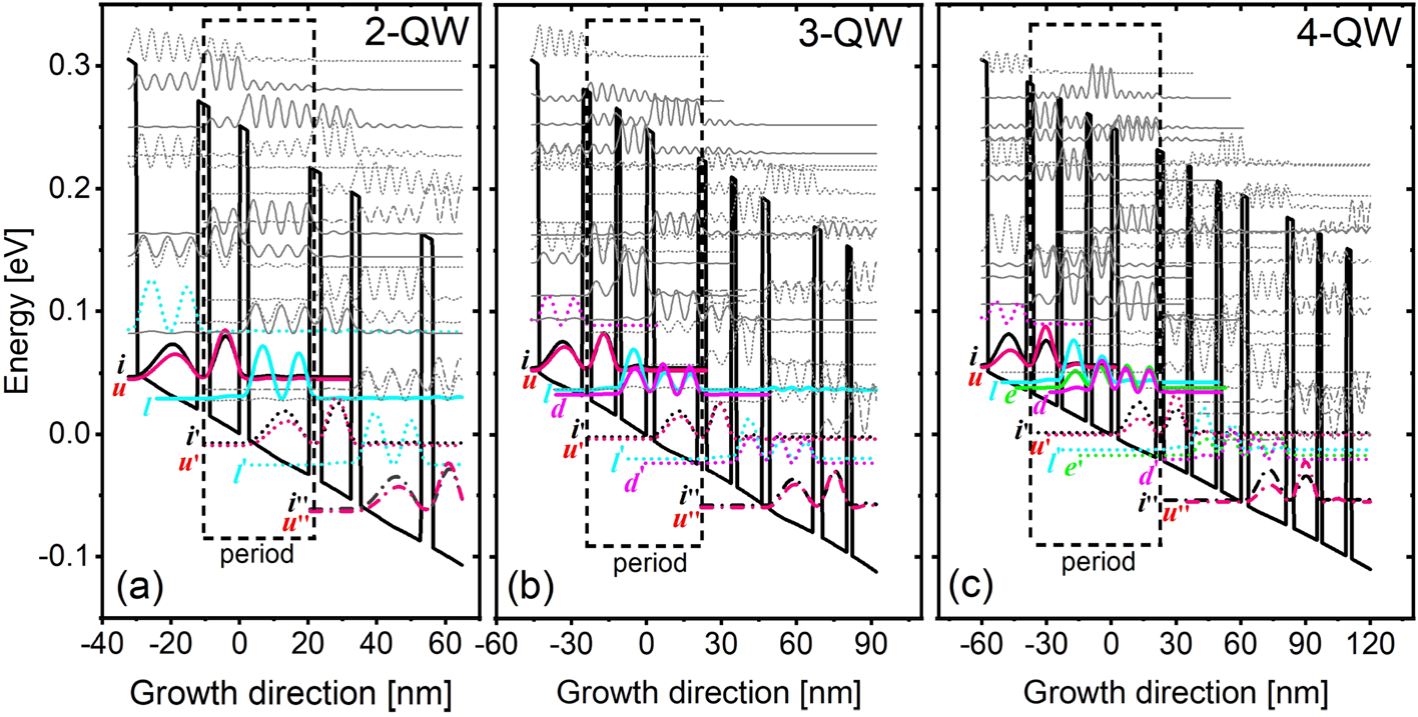
Conduction band diagram of three designs named as (**a**) 2-QW, (**b**) 3-QW, (**c**) 4-QW at operating bias. Moduli squared of the relevant electron wavefunctions within three neighboring periods are also shown. The thickness of stacked layers GaAs/Al_0.25_Ga_0.75_As (with a start from lasing barrier) and Si doping level(with doping position in the center 3 nm of widest QW) are shown, 2-QW: **2.5**/17.85/**3.4**/8.8 nm (doping level 5.5 × 10^10^ cm^−2^); 3-QW: **2.9**/17.7/**3.2**/10.1/**1.9**/10.4 nm (doping level 7.8 × 10^10^ cm^−2^); 4-QW: **2.9**/18/**3.1**/11.5/**2**/11.7/**2.27**/9.4 nm (doping level 1 × 10^11^ cm^−2^).

**Figure 3:**
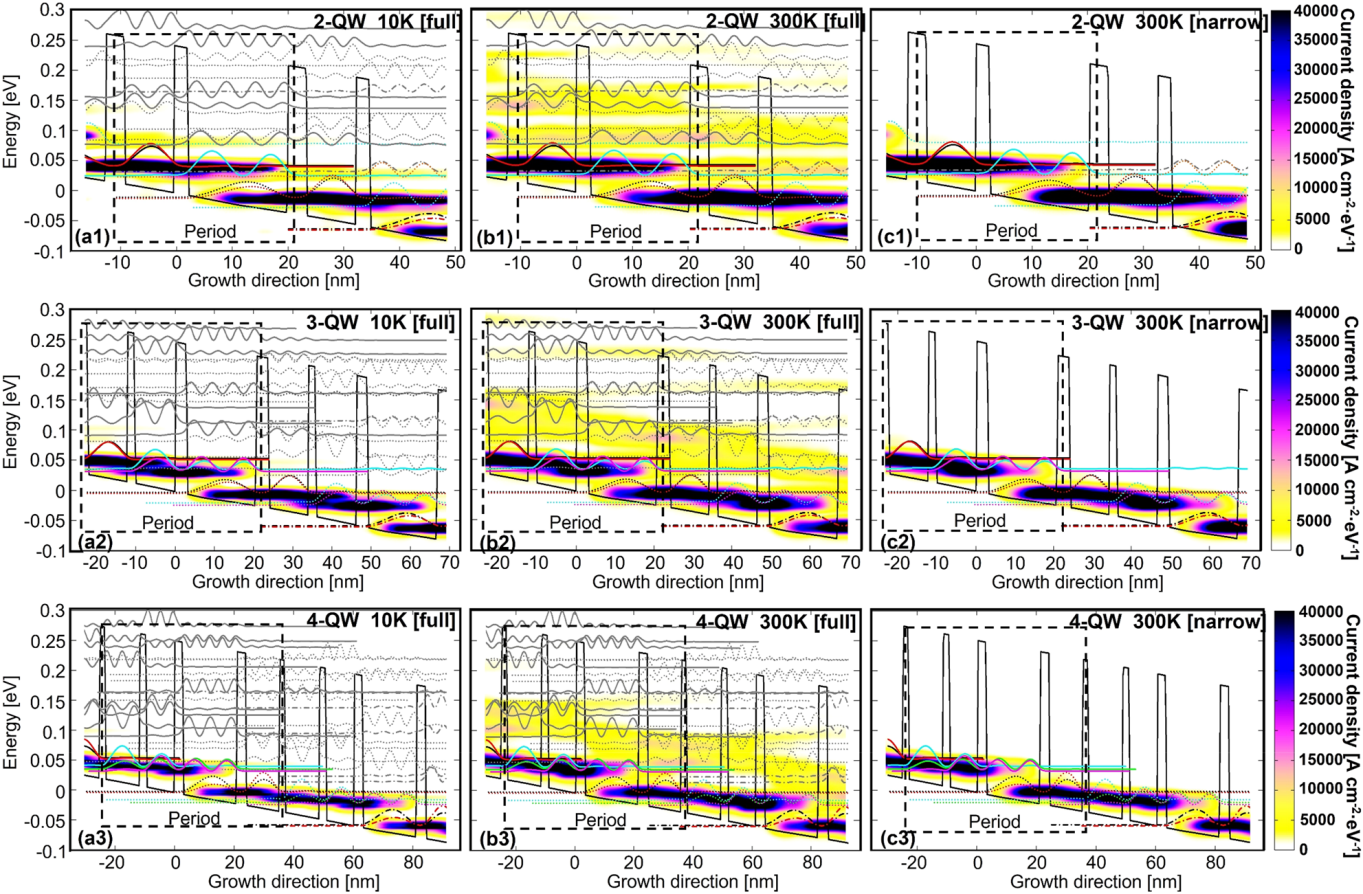
Energy and spatial resolved mappings of current distribution in 2-, 3-, 4-QW designs. (**a1**–**a3**) current mappings at 10 K with full-range (including all desired and high lying levels); (**b1**–**b3**) current mappings at 300 K with full-range; (**c1**–**c3**) current mappings at 300 K but with narrow-range (only including desired levels).

Figure 2 shows the three designs hereafter named as 2-QW, 3-QW, 4-QW. For 2-QW design (Fig. 2a), THz radiation is achieved relying on only three desired levels. When the operating bias is reached, the injection level ***i*** totally aligns with the upper laser level ***u***, electrons from the up-stream period can be injected into ***u*** by resonant-tunneling. Population inversion is formed between levels ***u*** and ***l*** (lower laser level) and emit THz photons through diagonal intersubband transition. Different from the interband lasers in which the population inversion can be naturally realized, a high depopulation efficiency of level ***l ***is essential for QCLs and requires careful engineering. For 2-QW design, the energy spacing of levels ***l*** and ***i′*** (injection level in the next period) is kept at ~ 36 meV (equal to LO phonon energy of GaAs) by engineering the thickness of lower well, thus the phonon emission will follow intrawell manner to empty the level ***l***. For 3-QW design (Fig. 2b), total four desired levels are employed in each period. The injection is same as in 2-QW design, but the depopulation of level ***l*** follows a different way, in which electrons are firstly extracted from level ***l*** into ***d*** via resonant-tunneling, then scatters down to level ***i′*** by vertical LO phonon emission. For 4-QW design (Fig. 2c), total five desired levels are used. The depopulation of level ***l*** is different from both 2- and 3-QW designs. Levels ***e*** and ***d*** act as minibands to firstly extract the carriers from ***l*** via strong elastic scatterings, a following relaxation process is same as the other two design, that is, electrons move down from level ***d*** to ***i′*** based on intrawell phonon emission. Although 4-QW design relies on more complicated QWs structure, it indeed has high tolerance toward the thermal backfilling^24^ and also the variation of layer thickness^25^. For those three designs, it is summarized here: (1) The periodic length: 2-QW(32.55 nm) < 3-QW(46.2 nm) < 4-QW(60.87 nm). If the laser frequency is expected at 3.7THz in all three designs, the periodic operating bias will be roughly equal to 55 mV (simply estimated by ~ (*E*_photon_ + *E*_phonon_)/*q*). As a result, the electric field strength of 2-QW is strongest, secondary in 3-QW, least in 4-QW; (2) By using tall barriers (Al% in AlGaAs with 25%), the number of confined high lying levels: 4-QW > 3-QW > 2-QW, it is easy to observe them in Fig. 2; (3) The high lying levels are quite close to the desired levels. Those features will lead to quite complicated leakages, especially at high temperature.

Figure 3 shows the energy and spatial resolved current mappings under the operating bias. The cut-off range of lateral energy is intentionally controlled: a full range indicates an energy range fully extended to the continuums, which means all desired and high lying levels are included in the calculations. The narrow range represents only desired levels, all high lying levels and also the continuum are excluded. By doing this, clear pictures of leakages through high lying levels can be obtained. With the full-range condition (Fig. 3a1–a3,b1–b3), when the temperature increases from 10 to 300 K, the current clearly flows out the desired levels and enters into high lying levels (even continuum area) in all three designs. It is immediate to identify a critical role of high lying levels for those thermally activated leakages. The narrow-range current mappings at 300 K (Fig. 3c1–c3) provide further evidence that leakages almost disappear if high lying levels are intentionally excluded.

The status of leakages is quite different in those three designs: (1) Even at low temperature of 10 K, the *x*-axis leakages (along the growth direction) extending into down-stream periods are more serious in 2-QW design. This type of leakage originates from desired levels, as shown in Fig. 3a1 (yellow bar). Thanks to the narrower period length and stronger electric field in 2-QW design, the high lying levels are much lower in energy. This will result in complicated level couplings between neighboring periods, and forms *x*-axis leakage channels. The thermally activation of electrons is weak at 10 K, so the *x*-axis leakages are mainly ascribed to sequential tunneling by elastic scatterings. (2) At a high temperature of 300 K, *x*-axis leakages are enhanced (Fig. 3b1–b3, orange bar). Meanwhile, *y*-axis leakages (in energy scale) are emerging (Fig. 3b1–b3, yellow colors). As compared with 2- and 3-QW designs, the *y*-axis leakages in 4-QW are relatively weak. This type of leakages is mainly caused by the thermally activated up-scatterings, as the electron–phonon absorption rate becomes larger when temperature increases.

**Figure 4:**
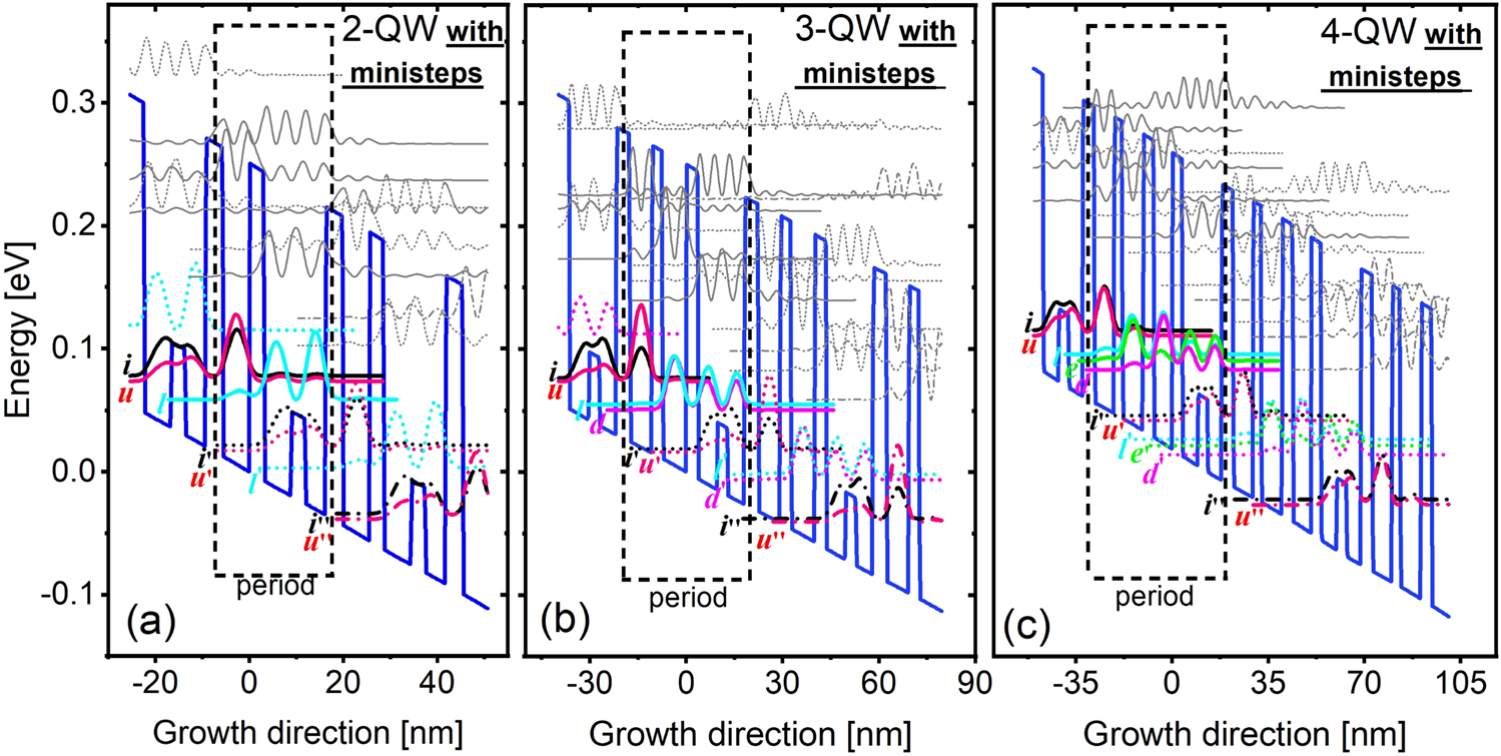
Conduction band diagram and quantum levels in (**a**) 2-QW, (**b**) 3-QW, (**c**) 4-QW with ministep at operating bias. The thickness of stacked layers GaAs/**Al**_**0.25**_**Ga**_**0.75**_ (with a start from lasing barrier) and Si doping level(with doping position in the center 3 nm of widest QW) are shown, 2-QW(ministep **Al**_**0.06**_**Ga**_**0.94**_**As**): **3.1**/13.1(***3***)/**3.7**/5.59 nm(doping level 5 × 10^10^ cm^−2^); 3-QW(ministep **Al**_**0.06**_**Ga**_**0.94**_**As**): **3.6**/14.5(***3.3***)/**4.3**/6.76/**3.3**/7.44 nm (doping level 7.82 × 10^10^ cm^−2^); 4-QW(ministep **Al**_**0.06**_**Ga**_**0.94**_**As**): **3.4**/14.4(***3.5***)/**4.05**/7.1/**2.73**/7.51/**3.8**/6.6 nm (doping level 9.73 × 10^10^ cm^−2^).

**Figure 5:**
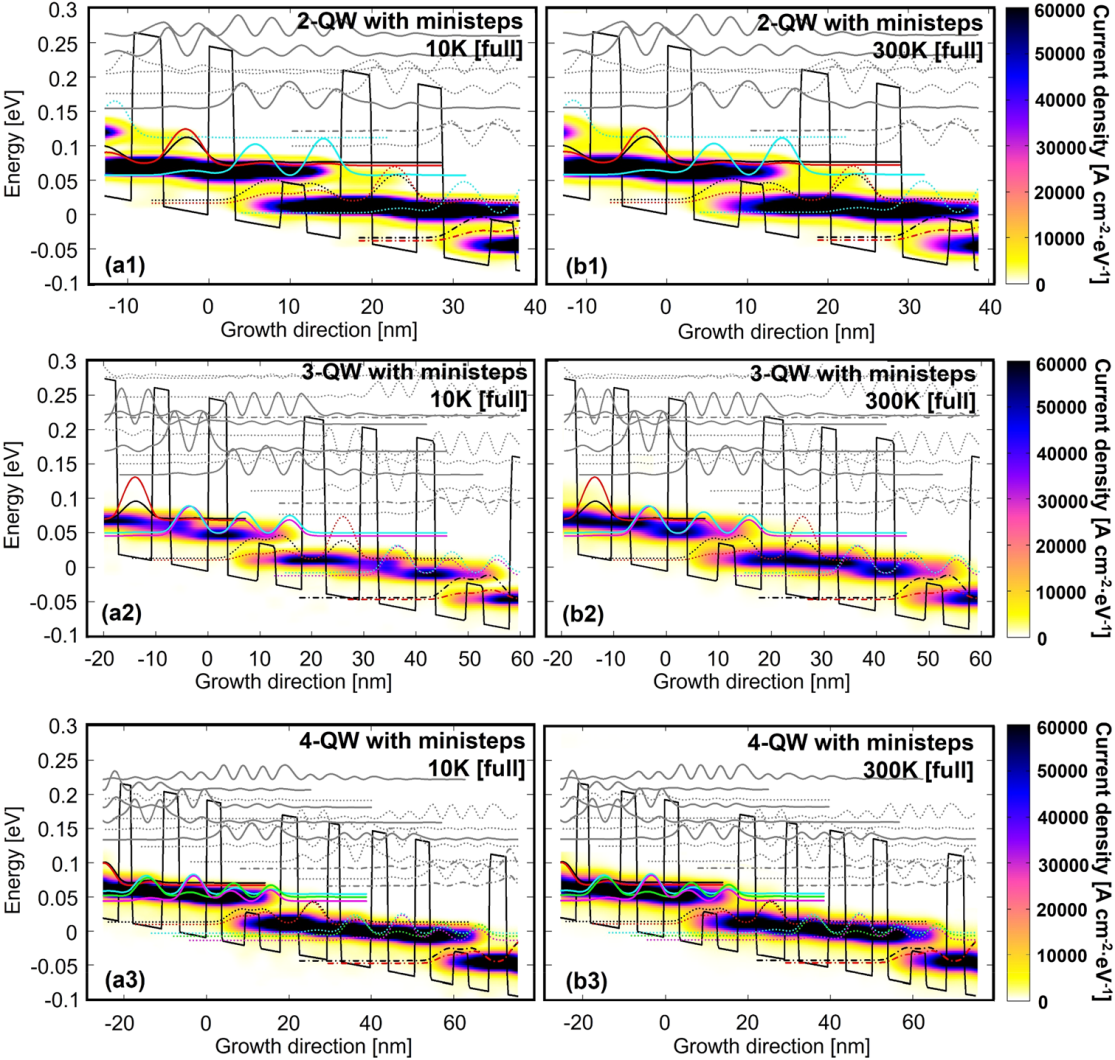
Energy and spatial resolved mappings of current distribution in three ministep designs with full-range at low/high temperature of 10 K/300 K (**a1**,**b1**: 2-QW with ministep; **a2**,**b2**: 3-QW with ministep; **a3**,**b3**: 4-QW with ministep).

With purposes to block those leakage channels, we introduce ministep to isolate the desired levels. Figure 4 plots the designs with ministep. The ministep (low barrier with Al_0.06_GaAs) is positioned at the center of widest QW. In such a way, high lying levels in this QW can be moved upward. To compensate the change of this QW, the other QWs also need to be narrowed correspondingly, thus the high lying level in all QWs are moved upward. If we directly compare the designs in Figs. 2 and 4, high lying levels area is clearly separated from the desired levels by using ministep. For radiation process, the designs with ministep are totally same as the original ones in Fig. 2. The current mappings of 300 K are shown in Fig. 5. With full-range, both *x*- and *y*-axis leakages are almost suppressed, the current distribution is nearly same as compared with the narrow-range (without high lying levels). The feasibility of ministep in experiment has been initially verified based on 2-QW design.

**Figure 6:**
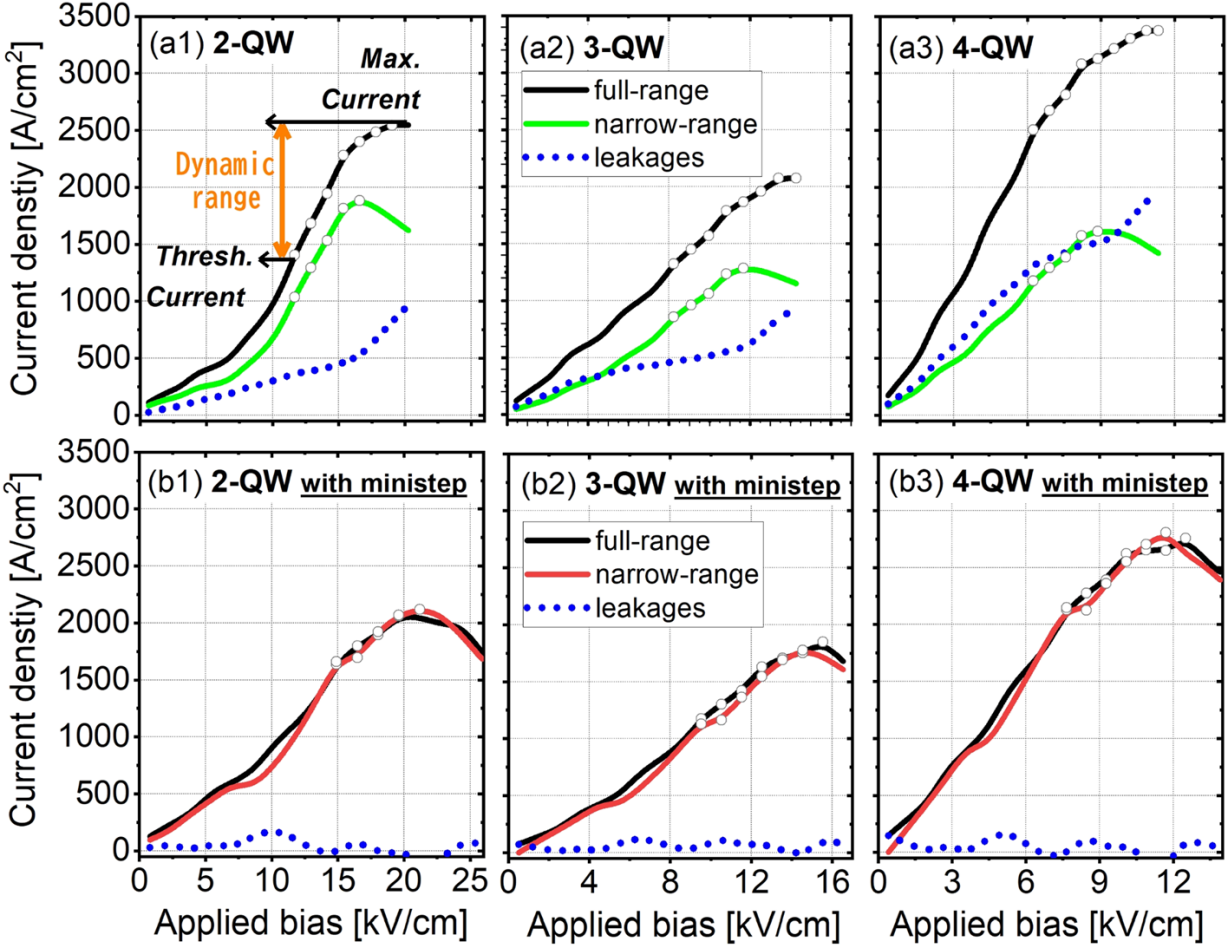
Current–voltage (I–V) curves and leakages through high lying levels as functions of applied bias for both original and ministep designs at 300 K. (**a1**–**a3**) 2-,3-,4-QW original designs and (**b1**–**b3**) 2-,3-,4-QW ministep designs with both full- and narrow-range. Meanwhile, the current leakages via high lying levels are also plotted. In addition, the dynamic range in each case is marked by hollow circles.

From I–V curves in Fig. 6, further quantitative comparations at 300 K are shown. (1) Leakages at 300 K: Under the full-range condition, for all three original designs (Fig. 6a1–a3, black curves), when the bias across periodic QWs exceeds the operation point, the current curves keep climbing further. It is because that, if higher bias applied, the high lying levels are lower in energy and couple stronger with the desired levels, thus leading to more serious leakages. In contrast, if the high lying levels excluded, the I-V plots actually begin to bend down (Fig. 6a1–a3 with the narrow-range, green curves) and enter into the negative differential resistance (NDR) region. The reason for NDR appearance in narrow-range condition is, the resonant-tunneling between levels ***i*** and ***u*** will degrade due to the misalignment of them at a higher applied bias. As a result, the current curves are overturned. No NDR region in full-range case is due to a compensation that high lying levels can contribute more currents (leakages). The current leakages are quantified as following: at the operating bias, the current leakages in 2-, 3- and 4-QW original designs are 500 A/cm^2^, 600 A/cm^2^ and 1500 A/cm^2^. It is even 3 times in 4-QW design as compared with the 2-QW one. The leakages possess a fraction of total current with 21%, 30%, 50% respectively. The reason of stronger leakages in 4-QW design is, this design has more high lying levels and can form more leakage channels. By using the ministep, we can observe the clear NDR in I–V plots even in case of full-range (Fig. 6b1–b3). The plots are almost coincident with each other no matter the high lying levels included or not (black curves for full-range; red curves for narrow-range). It can be explained here, after the high lying levels moving upward, even at higher bias, the high lying levels are still positioned far away from the desired levels. It confirms that the desired levels are truly isolated by using ministep. (2) Dynamic range: The current leakages can largely influence the dynamic range. As discussed in Ref.^26^, the lasing behavior basically requests a positive dynamic range in I-V plot. We also point out this dynamic range (the arrows shown in Fig. 6a1). Larger of this range, better for lasing in experiments. We draw this range with hollow circles in the I-V plots of Fig. 6. Note that the threshold current here represents a start of positive population inversion (not lasing threshold). The dynamic ranges in 2-QW, 3-QW and 4-QW original designs under a full-range are 1168A/cm^2^, 740A/cm^2^ and 877A/cm^2^, the corresponding values are 496A/cm^2^, 701A/cm^2^ and 634A/cm^2^ in the ministep designs. It is obvious that ministep designs are with smaller dynamic ranges but showing different changes: this range only slightly decreases for 3- and 4-QW but largely reduces even ~ 50% for 2-QW. The 2-QW design is actually quite attracting to realize high-temperature operations, due to its minimum number of desired levels. Higher optical gain is expected due to the much narrower periodic length. As discussed in this work, the 2-QW design benefits more from isolated level system by using ministep as its original design has the strongest leakages. However, the shrinking of dynamic range will bring more difficulties in real growth. Any variations of layer thickness in experiments may further reduce this range even quench the lasing.

## Conclusion

In summary, based on the most used THz-QCL designs, it is found that the thermally activated leakages are common issues but closely depends on the detailed design. The leakages even possess a fraction more than 50% of the total current in 4-QW design. Those leakages pass the high lying levels in both *x*- and *y*-axis directions. The *x*-axis leakages originate from direct tunneling between high lying and desired levels, even extending across several neighboring periods. The *x*-axis leakages are activated even at a low temperature of 10 K. The *y*-axis leakages are ascribed to thermally activated up-scatterings from injection/upper laser levels into the high lying levels. We introduce ministep in the widest QW, all the high lying levels then can be moved upward by narrowing all QWs. The desired levels can be well isolated, thus both the *x*- and *y*-axis leakages are effectively blocked.

## Method

The current distributions through levels are quantitatively calculated using the non-equilibrium Green’s function (NEGF) formalisms^28–31^. It basically solves the four coupled partial differential equations, which are expressed in operator forms:

1 $$({\text{E}} - {\text{H}}_{0} {-}{\text{ e}}\Phi - \Sigma^{{\text{R}}} ) \text{G}^{{\text{R}}} = 1$$

2 $${\text{G}}^{ < } = {\text{ G}}^{{\text{R}}} \Sigma^{{\text{R}}} {\text{G}}^{{{\text{R}}\dag }}$$

3 $$\Sigma^{ < } = {\text{ G}}^{ < } {\text{D}}^{ < }$$

4 $$\Sigma^{{\text{R}}} = {\text{ G}}^{{\text{R}}} {\text{D}}^{{\text{R}}} + {\text{ G}}^{{\text{R}}} {\text{D}}^{ < } + {\text{ G}}^{ < } {\text{D}}^{{\text{R}}}$$

where H_0_ is the electronic Hamiltonian of a single charge carrier, Φ is the electrostatic potential, D is the sum of all environmental Green’s functions, and Σ denotes the self-energy. Those expressions are implemented in real space basis. The scattering states, the transition probabilities between them and also their occupations are calculated within self-consistent manners. The electric boundary condition is considered. For each scattering forms, inelastic (electron–phonon interactions) and elastic (charged impurity, interface roughness, alloy disorder) scatterings are included. Also, the electron–electron interactions are considered^31^. More details of scatterings are described in Refs.^30,31^. In contrast to the NEGF studies in previous reports^32,33^, the *in*-plane momentum dependence of the scattering matrix element is employed. For the magnitude of leakages, we tune the cut-off energy with/without the high lying levels, and simply estimate the differences of the total current.
